# Feasibility and Acceptability of a Text Message Intervention to Promote Adherence to Nutrition and Physical Activity Guidelines in a Predominantly Hispanic Sample of Cancer Survivors and Their Informal Caregivers: Results from a Pilot Intervention Trial

**DOI:** 10.3390/nu15224799

**Published:** 2023-11-16

**Authors:** Melissa Lopez-Pentecost, Sophia Perkin, Sarah Freylersythe, Paola Rossi, LaShae D. Rolle, Sara M. St. George, Tracy E. Crane

**Affiliations:** 1 Sylvester Comprehensive Cancer Center, University of Miami Miller School of Medicine, Miami, FL 33136, USA; 2 Mel and Enid Zuckerman College of Public Health, University of Arizona, Tucson, AZ 85724, USA; 3 Department of Public Health Sciences, University of Miami Miller School of Medicine, Miami, FL 33136, USA; 4 Medical Oncology, University of Miami Miller School of Medicine, Miami, FL 33136, USA

**Keywords:** mHealth, diet, exercise, survivorship, caregivers, Latinos, Mexican

## Abstract

Hispanic cancer survivors face unique barriers to meeting American Cancer Society (ACS) nutrition and physical activity guidelines, which reduce the risk of cancer recurrence and mortality and improve quality of life. This pilot intervention trial evaluated the feasibility and acceptability of a two-week ACS guideline-based nutrition and physical activity text message intervention in a predominantly Hispanic sample of cancer survivors and their informal caregivers. A mixed methods approach was used to assess feasibility and acceptability of the intervention. Feasibility and acceptability were measured by meeting a-priori cut-offs of >80% for recruitment, retention, and text message response rate. Participants also completed a semi-structured exit interview by telephone that assessed intervention components. Thirteen cancer survivors and six caregivers (*n* = 19) participated in this pilot study; 78% self-identified as Hispanic. Mean time since treatment completion for survivors was 11.9 years (SD 8.4), and 67% had breast cancer. Cancer survivors had a higher acceptability rate for physical activity (94%) compared to nutrition messages (86%), whereas equal acceptability rates were observed for both types of messages among caregivers (91%). Texting interventions are a feasible, acceptable, and a cost-effective strategy that have the potential to promote lifestyle behavior change among Hispanic cancer survivors and caregivers.

## 1. Introduction

Advancements in cancer screening and treatment in the United States (US) have led to significant improvements in early detection and timely, effective treatment. As a result, the number of cancer survivors continues to increase with an estimated 18 million survivors as of 2022 [1]. While these advancements are a win for cancer, a plethora of challenges and considerations remain which have a substantial impact on cancer burden. Reports indicate that while racially and ethnically diverse populations in the US, such as the Hispanic population, have a lower incidence of cancer compared to other racial and ethnic heritages, cancer remains the leading cause of death among this group [2]. Hispanic cancer survivors experience increased odds of late-stage diagnosis, are less likely to receive definitive treatment, and have worse odds of cancer-specific and overall survival compared to non-Hispanic Whites and Asians, respectively [2,3]. Additionally, research shows that Hispanic cancer survivors have high rates of comorbid conditions after cancer treatment that may further exacerbate lingering symptoms and worsening quality of life after care [4,5,6]. The continued lack of representation of the Hispanic community in research hinders the ability to develop and implement equitable and effective cancer prevention and control strategies aimed at reducing the cancer burden of this population [7].

The American Cancer Society (ACS) has set forth nutrition and physical activity guidelines for cancer survivors that emphasize the promotion of consuming a dietary pattern rich in a variety of plant foods (such as vegetables, whole fruits, whole grains, and beans and legumes), excluding or limiting consumption of red and processed meats, sugar-sweetened beverages, highly processed foods, and refined grain products, and engaging in at least 150 min of moderate to vigorous physical activity per week [8,9]. Greater adherence to these ACS guidelines is associated with reduced risk of cancer recurrence and mortality [8,9,10,11], and improved quality of life [12]. However, reports estimate that only 29% and 43% of Hispanic cancer survivors meet nutrition and physical activity recommendations, respectively [13].

A number of behavioral and supervised lifestyle interventions have been developed to promote improvements in diet and physical activity behaviors among cancer survivors [14]. However, well-established barriers affecting engagement of the Hispanic community are noted in such interventions, including transportation, lack of childcare, and work/time conflicts [7,15]. On the other hand, cultural adaptations such as the inclusion of family members as part of the intervention have been reported as effective for research engagement [16,17,18]. In fact, increased family and community support was highlighted as one of five strengths of culturally adapted interventions in a recent report of interventions among racial and ethnic minorities [19]. Particularly, previous lifestyle and psychosocial interventions among Hispanic women cancer survivors show that strong family ties play a significant role in providing survivors with emotional, social, and functional support [17,20], with interventions targeting social support and inclusion of caregivers being linked to improved outcomes such as higher quality of life [17].

Interventions that incorporate culturally-relevant components, including social support, such as the inclusion of a caregiver, combined with digital health tools, such as text messaging, mobile Health (mHealth) apps, and telehealth, present as promising strategies to enhance interventions and overcome self-reported barriers to participation in lifestyle interventions for Hispanic cancer survivors. Intervention delivery approaches that include digital health tools, particularly messaging, may hold heightened potential for the Hispanic community who have the highest texting, cell phone ownership and use rates than any other racial and ethnic groups [21,22]. Additionally, health care delivery underwent a digital transformation that was accelerated by the COVID-19 pandemic, which fundamentally altered medical care and reach [23]. Although studies testing the efficacy of digital lifestyle interventions among the Hispanic population and their caregivers are limited, a few studies developed for Hispanic breast cancer survivors have yielded promising results related to acceptability and satisfaction with the use of digital health tools [20,24]. However, further investigation into the adequate implementation of mHealth modalities such as texting, and the texting content, is needed to understand how to effectively apply such methodologies in future interventions. Moreover, the benefit of incorporating a caregiver in such intervention studies is twofold. First, by receiving the same messages as survivors, caregivers are aware of the information shared with the survivor thus providing an opportunity for support to promote the intended behavior changes. Secondly, caregivers similarly benefit from the content, as the text messages provide information that promote cancer preventive behaviors. Therefore, including survivors and caregivers represents a promising strategy likely to be adopted in the development of future intervention studies for this population [17,19,20]. While previous digital health interventions for Hispanic cancer survivors and their caregivers have been conducted [24,25,26,27], to our knowledge, acceptability of ACS nutrition and physical activity-based text-messaging content has not been evaluated. To address these gaps, the aim of this pilot intervention trial was to evaluate the feasibility and acceptability of a two-week ACS guideline-based nutrition and physical activity text message intervention and message content in a sample of cancer survivors and informal caregivers who closely mirrored the demographic composition of the catchment area of The University of Arizona Cancer Center, covering the entirety of southern Arizona and consisting predominantly of Hispanic ethnicity.

## 2. Methods

### 2.1. Study Sample

This pilot intervention trial took place between September 2018 and February 2019. A sample of cancer survivors and individuals who self-identified as their informal caregivers, defined as individuals in the survivor’s social network, related by blood or social attachment, who provide emotional, informational, and/or instrumental support were enrolled in the study [28]. To be eligible for the current study, participants had to be 21 years of age or older, living in Southern Arizona, and have a US cell phone number with text message capability. Recruitment efforts included reaching out to individuals who had previously provided their consent to be contacted for potential future research. Recruitment strategies also involved word of mouth and flyers in a local community in Tucson, AZ. Although recruitment focused on enrolling individuals from Hispanic ancestry, enrollment was extended to any eligible cancer survivor or self-identified caregiver who showed interest and could benefit from participating in the study. Individuals enrolled mirrored the demographic composition of the catchment area of The University of Arizona Cancer Center (UACC) in Southern Arizona. Cancer survivors’ caregivers were invited to participate in the study though they were not required to enroll as dyads. Participants previously completed a baseline questionnaire prior to initiation of the intervention to gather information on demographic characteristics.

### 2.2. Intervention

**Figure 1:**
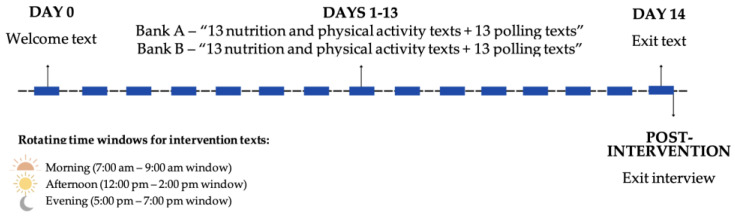
Texting intervention study timeline.

As part of this two-week pilot intervention study, participants received ACS guideline-based nutrition and physical activity text messages. Two different messaging banks were developed to test a greater variety of message content. Participants were assigned to receive messages from Bank A or Bank B in their preferred language (*n* = 13 nutrition and *n* = 13 physical activity messages in each bank) by study coordinator using an alternate allocation method. Participants were not informed which bank of text messages they were assigned to but the interventionist who oversaw the trial delivery were informed for logistic purposes as text messages were scheduled to be sent ahead of time. While the text messages did not specifically outline the exact ACS guidelines, messages in both banks focused on nutrition and physical activity tips and recommendations that would promote behaviors and would collectively contribute to meeting such guidelines. The messages were purposely developed to be under 160 characters in length. Over a two-week period, participants received a welcome and an exit text (*n* = 2), 26 intervention texts (one nutrition and one physical activity daily), and 26 polling texts (one following each intervention text; Figure 1). The purpose of each polling message was to assess acceptability; participants responded with a “1” if the message was rated as useful or a “2” if the message was rated as not useful. Each message was automated and scheduled to be sent during one of the three different time windows using a rotating schedule. The time windows were as follows: (1) Morning; 7:00–9:00 a.m., (2) Afternoon; 12:00–2:00 p.m., and (3) Evening; 5:00–7:00 p.m. All messages were adapted from a previous lifestyle intervention [29] by trained lifestyle behavioral interventionists using a culturally targeting approach [30] for the demographic composition of the catchment area of the UACC, consisting predominantly of Hispanic ethnicity. Messages were reviewed by a community advisory board of Hispanic cancer survivors who provided feedback on content including best practices for addressing nutrition and exercise in the community. The intervention was overseen by two lifestyle behavior health coaches with training on diet and physical activity. Health coaches had more than four years of health coaching experience for changing lifestyle behaviors and specific experience with cancer survivors. Strategies implemented in the content development of the text messages included linguistic (i.e., text messages provided in the preferred language of the participant), constituent-involving (i.e., including bilingual and bicultural staff in the development of the text messaging content and conducting the translations), and sociocultural (i.e., providing nutrition and physical activity tips and information based on cultural foods and practices). Additionally, the text messages utilized a theoretical framework based on the COM-B model for behavior change which considered capability (C), opportunity (O), and motivation (M) as three key factors capable of inducing change in behavior (B) [31]. The texting intervention aimed to provide low-maintenance and low-burden tips for behavior change that could be easily incorporated into this target population’s daily routine. Three quarters of the text messages were targeting capability and opportunity, while the remaining text messages were motivational in attempt to achieve behavior change (Supplementary Tables S1–S4 lists the text messages as well as corresponding response and acceptability rates).

### 2.3. Feasibility and Acceptability

Feasibility and acceptability of the intervention were assessed using a mixed methods approach of quantitative and qualitative data. Feasibility was assessed by meeting a-priori cut-offs of ≥80% on several parameters, including: (1) number of participants who enrolled in the study out of those who were eligible (recruitment); (2) number of participants who completed the intervention (retention); and (3) number of polling messages participants responded to out of the total number of messages they received (response rate). Intervention acceptability was measured through the polling texts sent immediately after each of the daily nutrition and physical activity texts that were responded with a “1” indicating the text was found to be useful to the participant, out of the total number of responded poll texts. Acceptability of the text messages in Bank A vs. Bank B were not compared against each other as the purpose of intervention was to examine overall acceptability of the intervention as well as the content of the text messages individually. Both feasibility and acceptability of the intervention were further assessed qualitatively at the end of the intervention through semi-structured exit interviews conducted over the phone by two trained graduate-level bilingual and bicultural research assistants in either English or Spanish based on participant preference. Topics discussed in the interviews included dosage and timing of texts, texting modality satisfaction, content preferences, behavior change, and cultural appropriateness of the intervention messages (Supplementary Table S5 summarizes the exit qualitative interview questions and themes). Participants consented to having their interview audio recorded, and the recordings were then transcribed and reviewed for accuracy by the same individuals who conducted the interviews. Spanish transcripts were not translated to English to preserve meaning and context. Using Braun and Clarke’s six phases of thematic analysis [32], each exit interview was reviewed and analyzed separately by the same two research assistants using Microsoft Word. As part of the analytic process, each reviewer first immersed themselves in the data by reading and re-reading each transcript while annotating with preliminary semantic codes and noting any developing subthemes. Next, each reviewer engaged in a systematic and independent coding process by reviewing each transcript and using semantic codes to capture explicitly-expressed meaning in the data. Next, the reviewers compared codes and notes and discussed any discrepancies until a final set of codes was developed. While a formal metric for inter-rater reliability was not obtained, given the semantic nature of the coding, a high level of agreement was found between the reviewers who both captured and coded similar ideas. The two reviewers then met again to organize similar codes within each pre-determined theme and together, generated salient sub-themes.

This study was conducted in agreement with the ethical standards specified in the 1964 Declaration of Helsinki and its later amendments and was reviewed and approved by the University of Arizona Human Subjects Protection Program (Institutional Review Board #1808895982). Informed consent was obtained from all participants included in the study and upon signing, all participants received a copy of the consent. All data were de-identified for privacy and confidentiality purposes. Participants who completed the intervention and exit interviews were compensated with a $25 gift card to a local chain retailer.

## 3. Results

### Baseline and Demographic Characteristics

Demographic characteristics of participants enrolled in the study are summarized in Table 1. Mean age was 62.5 (±12.4, range 44–90) years for survivors and 53.2 (±18.5, range 25–75) years for caregivers. For cancer survivors, years since diagnosis was 11.9 (±8.4) on average. The study sample was composed of 78% Hispanic (*n* = 14), 5% non-Hispanic Black (*n* = 1), and 17% non-Hispanic White (*n* = 3).

## 4. Quantitative Outcomes

### 4.1. Feasibility

**Figure 2:**
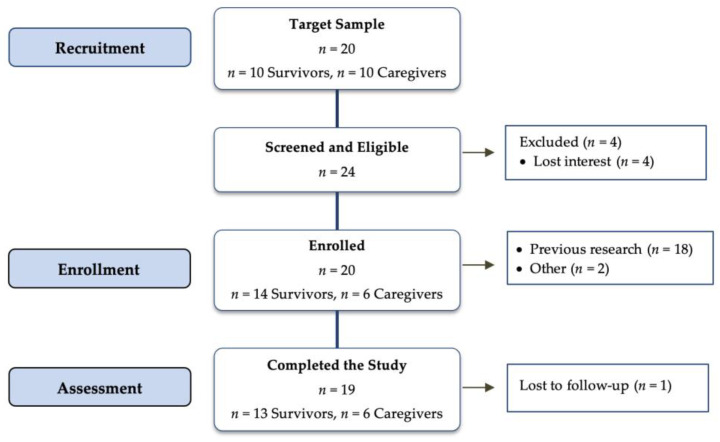
Consort flow diagram.

Target sample for this study was 10 survivors and 10 caregivers (*n* = 20). Participants were recruited from a list of individuals who consented to be contacted for future research, and secondarily through word of mouth, flyers, and community events. Of the 24 participants invited to participate, 20 (83%) were enrolled (14 cancer survivors and 6 caregivers), and 19 completed the study (95%) (Figure 2). On average, participants responded to 24 of the 26 polling messages that followed each nutrition and physical activity message received (92%) (Table 2). Cancer survivors had an overall higher response rate to the follow-up polling messages compared to caregivers (97% vs. 81%, respectively). Cancer survivors had a 1% higher response rate to nutrition messages (98%) compared to physical activity (97%), whereas caregivers had a 3% higher response rate to physical activity messages (82%) compared to nutrition (79%).

### 4.2. Acceptability

Acceptability was determined by the number of messages rated as “useful” from the polling messages to which participants responded (Table 2). Acceptability for both types of messages combined was 90%, with physical activity messages having a 6% higher acceptability than nutrition messages (93% vs. 87%) in the total study sample. Among cancer survivors, physical activity messages had an acceptability rate 8% higher than nutrition-related messages (94% vs. 86%, respectively), whereas among caregivers, acceptability rates were the same for both types of messages (91%).

There were 8 participants (*n* = 6 survivors, *n* = 2 caregivers) randomized to Bank A, and 11 participants (*n* = 7 survivors, *n* = 4 caregivers) randomized to Bank B. Overall, 85% of the messages in both Banks A and B (44 out of 52), had an acceptability rate greater than 80%. In Bank A, 9 out of 13 (69%) nutrition messages and 11 out 13 (85%) physical activity messages had acceptability rates above 80% (Supplementary Tables S1 and S2). In Bank B, 92% of both nutrition and physical activity messages (12 out of 13) had acceptability rates above 80% (Supplementary Tables S3 and S4).

## 5. Qualitative Outcomes

A total of 17 participants completed the qualitative exit interviews (13 survivors and 5 caregivers). Qualitative findings for caregivers mirrored the major themes identified among survivors. Therefore, to avoid redundancy and overinterpretation of results by stratification of a limited sample size, qualitative findings for the total study sample, survivors and caregivers, are presented together. Below, we describe salient subthemes generated within each of the overarching topics of the interviews (Supplementary Table S5 summarizes the exit qualitative interview questions and themes).

### 5.1. Intervention Dosage and Timing

#### Dosage and Timing of Text Messages Were Adequate

Regarding intervention dosage, participants reported that the number of messages per day (two) was not burdensome. Participants described looking forward to the next message, and they shared that the quantity was adequate to promote behavior change:

*“I don’t think you want to be bombarded. It is almost like if you only got one it would not be enough, but two, I don’t know, it seems just right.”*

—Participant 305a (Survivor)

*“I kind of looked forward to them and every so often I checked to make sure I got them. But yeah, I think two a day is a good number.”*

—Participant 301b (Caregiver)

Similarly, most participants agreed that receiving messages during the three established time windows (morning, afternoon, and evening) were acceptable and convenient, and although a few participants mentioned the morning time was not ideal (7:00–9:00 a.m.), they felt it was not too burdensome as they could read the messages and reply at a time convenient for them. One participant mentioned that the messages aligned with their daily schedules while another one reported the morning messages were sent too early in the morning:

*“[the time I received the messages] seemed fine to me, they were sent to me at a very good time because I get up early in the morning, so I didn’t have any problems.”*

—Participant 901a (Survivor)

*“Sometimes I received the messages very early in the morning, like 8, but it was fine because I answered them when I could so there was no problem.”*

—Participant 342a (Survivor)

### 5.2. Texting Modality Satisfaction

#### Texting Was Flexible and Convenient

Participants reported satisfaction with the choice of utilizing text messaging as the modality to receive lifestyle-related information. Participants described this modality as an easy and accessible way to receive information that can be accessed at any time. Some described their preference for texting over phone calls or the internet with one participant expressing feeling a sense of freedom to participate in this intervention due to the high flexibility of the texting modality.

*“What I like about text in all environments is that you can answer it in your own time per say. So, it is not as you know, a phone call comes in and you go “ugh I can’t take that right now”. With a text … it is not as intensive communicating, or as stressful as other forms of communications. You know, you look at it, read, it and give an answer, so it works out well.”*

—Participant 301a (Survivor)

*“It was perfect. You know, I want to say that this was the study that I have enjoyed the most just because I had the freedom of getting to it when I had the time or getting to it later and I never felt pressured and all the tips, very, very cool.”*

—Participant 332a (Survivor)

*“Although I am technologically literate, I do not have internet at home, so I do appreciate being able to get the text messages without having to go to the library and look at my email.”*

—Participant 301b (Caregiver)

### 5.3. Intervention Content Preferences

#### Desire for more Educational Information

While participants described the overall content of the intervention as being useful and interesting, they particularly highlighted their preference for educational messages that linked nutrition or physical activity to specific biological mechanisms and health.

*“The one I particularly liked was the one about fruit and green leafy vegetables having antioxidant and their role in cancer, as a cancer survivor, I really liked this topic.”*

—Participant 901a (Survivor)

*“I would suggest topics like foods we eat and their effects on the body. For example, when you eat sugars and it can lead to diabetes, and how diabetes can lead to kidney disease, and it is all a domino effect.”*

—Participant 325a (Survivor)

When participants were asked to provide suggestions for intervention content, they requested more information about how to manage long-term cancer side effects, particularly nausea and difficulties with sleep, and the potential role of lifestyle to manage them.

*“I have nausea after my cancer and I still deal with that, so maybe other patients are dealing with it too. Maybe there can be things with how to deal with the nausea, things like have ginger tea, you know, having an extra glass of water. You know those kinds of things would be very helpful.”*

—Participant 401a (Survivor)

*“One thing I find [interesting] is sleep. They can tell you to sleep but some people cannot sleep, they don’t address what to do when you can’t sleep.”*

—Participant 422a (Survivor)

### 5.4. Impact of Intervention on Self-Reported Behavior Change

#### 5.4.1. Intervention Reinforced Healthy Diet Behaviors

Participants reported the text messages were useful and liked the simplicity of the information. Some participants highlighted the reinforcing effects the messages had on their lifestyles, such as serving as reminders to drink water before meals and cooking at home when possible.

*“This whole thing was a good reminder …I think one of the reminders there was to drink a full glass of water before you eat, and I have been really good at doing that lately, but overall, this program was a good reminder too.”*

—Participant 301b (Caregiver)

*“I try to cook every day at home and do the best I can, but this [intervention] it’s a good reminder to take care of your health.”*

—Participant 325b (Caregiver)

#### 5.4.2. Intervention Led to Self-Reported Adoption of Healthy Physical Activity Behavior

Overall, messages that targeted capability and opportunity rather than those that used a motivational approach were perceived by participants as being more effective at promoting behavior change, particularly for physical activity behaviors. Participants reported that the messages which had a bigger impact on their physical activity were those that provided multitasking tips on incorporating physical activity during their daily responsibilities and activities:

*“There was one [message] that helped me a lot with leg stiffness…. that in itself was worth the whole thing. I have had that problem for years and now it has gone away.”*

—Participant 402a (Survivor)

*“I try to move around. Like when I brush my teeth, I just bend one leg and I stand on one leg and then the other. Just doing that it makes me realize that the stiffness is there… It feels good to actually move them and the knee is getting lubricated, and you are working some muscles in the leg. And it does not take any time!”*

—Participant 305a (Survivor)

*“I have tried to be conscious of multi-tasking with physical things and enjoying walking and this and that and making two trips instead of one, those kinds of things were useful.”*

—Participant 301a (Survivor)

### 5.5. Cultural Appropriateness

#### Intervention Content Was Perceived as Culturally Relevant, but Changes to Cultural Food Staples May Be Less Acceptable

Participants were asked about the cultural relevance and applicability of the messages. The majority of participants described the content to be easy to understand and, regardless of race and ethnicity background of participants, the intervention was considered universally relatable given diet and physical activity strategies to improve health are important for everyone. However, participants reported some hesitancy towards changing some cultural food staples:

*“The one about the tacos on the lettuce leaves, the part of the tacos, the corn tortilla, is what I really like. I think I can try your suggestion, but I don’t think I can sustain it.”*

—Participant 407a (Survivor)

*“The ones about diet, they were great but maybe it is a cultural thing, when it says to make a burrito with peanut butter and bananas, I don’t think burritos call for that. They usually have beans and cheese when you make them at home. That might not be the kind of thing I would have tried.”*

—Participant 305a (Survivor)

Other participants provided suggestions on how to further culturally target the message content given the Hispanic community are a heterogenous group and individuals from different countries of origin have various cultural food staples:

*“I was born and raised in Puerto Rico, and a lot of the meals suggestions that you mentioned in the study were geared toward people with a Mexican background… we Puerto Ricans eat a lot of rice and beans and I don’t know if something can be added geared towards other Latino communities.”*

—Participant 332a (Survivor)

## 6. Discussion

### 6.1. Principal Results

Hispanic cancer survivors and their informal caregivers need effective interventions to promote lifestyle change and improve their health. Participants in this pilot study who were predominantly Hispanic, English-speaking, college educated individuals, described the intervention as useful, non-intrusive, and effective at promoting some behavior change, particularly physical activity. Participants reported high satisfaction with the number and timing of the text messages and highlighted the advantages of utilizing texting as the intervention-delivery modality over other methods such as telephone calls or web-based. Participants considered the intervention to be relevant for everyone but provided insights on the cultural appropriateness of some nutrition messages that involved cultural food staples. Additionally, participants outlined specific intervention content they would benefit from in the future, including educational information on the specific biological effects of nutrition and physical activity, symptom management for cancer-related long-term side effects, and ways to increase the cultural relevancy of messages for the Hispanic community. Overall, quantitative and qualitative data show participants were more responsive to messages that targeted capability and opportunity rather than those that used a motivational approach as related to the COM-B model.

Results from the current study expand on findings from other mobile Health (“mHealth”) studies among cancer survivors, which altogether imply that several mHealth strategies are feasible and acceptable to this population. While no other study has focused on promoting ACS nutrition and physical activity guidelines in a pronominally Hispanic sample of cancer survivors and their caregivers, there is vast literature on mHealth-based studies designed to improve quality of life or other psychosocial symptoms in cancer survivors [33,34,35] with a few specific to the Hispanic population of cancer survivors [24,25,26,27]. These studies have successfully tested various mHealth delivery approaches including telephone calls [24,25], smartphone applications [26], online websites [27,36], email communication [27], or a combination of these [27,36], and have demonstrated their utility at addressing limitations of other conventional intervention approaches, particularly, barriers known to hinder participation of minority populations in intervention studies [16]. The current study expands on previous research with its findings that lifestyle-focused text messages grounded in the COM-B behavioral theory delivered twice per day demonstrate feasibility and acceptability in both survivors and their caregivers. These findings are also supported by Singleton et al., who tested a lifestyle-focused text message randomized controlled intervention trial among 387 breast cancer survivors [37]. Although the Singleton study did not focus on the Hispanic population or any minority populations nor include informal caregivers, participants reported that daily messages sent in the morning were acceptable, although very early morning were not preferred, but were not an inconvenience given they could access them any time, an advantage of using the texting modality [37]. In fact, in the current study, a big driver of acceptability was the advantage of utilizing the texting modality as the intervention delivery approach over other mHealth modalities. Participants described the current intervention as providing a sense of “freedom” compared to other modalities, such as telephone calls, where they feel pressured to respond immediately, or compared to web-based platforms that may create an extra challenge for participants who do not have internet at home. It is important to note that the current intervention study was designed as a preliminary pilot study to assess feasibility and acceptability and future, more rigorous trials with larger sample sizes may warrant a different or a combination of delivery modalities to successfully drive and assess the desired outcome.

Results from this study suggest that although acceptability of both nutrition and physical activity messages were high, participants had a greater preference for physical activity messages. In fact, participants reported that the intervention had a greater effect on their physical activity compared to eating behaviors, although formal assessment of these behaviors did not occur. These results have important implications given reports show that close to 50% of adult Hispanic women do not engage in any leisure physical activity, and leisure physical activity is lower among Hispanic women than in any other Hispanic heritage [38]. Participants described having a higher preference towards messages that targeted capability and opportunity rather than those employing a motivational approach. This finding was also supported by the quantitative data showing messages with tips on how to incorporate easy activities while completing other tasks, such as calf raises while brushing teeth, or extra trips when unloading groceries from the car or doing laundry, were highly rated, while lower acceptability rates were observed for messages that prompted participants to reflect on motivators for physical activity when motivation was particularly low. Promotion of physical activity behaviors in this study may have been facilitated by the fact that the physical activity recommendations did not require special knowledge or a high degree of self-efficacy for physical activity, both of which are among the top barriers for exercising in this group [38]. Additionally, it may be that incorporating a new habit, such as physical activity, is easier for individuals than replacing an existing one, such as dietary habits, which may take additional effort and consideration given cultural and familial influences [39,40].

The study design and intervention content were utilized linguistic, constituent-involving, and sociocultural strategies to culturally target the Hispanic population of Southern Arizona. Overall, participants considered the intervention to be adequate for anyone, given lifestyle and human health are global aspects applicable for all. However, quantitative and qualitative results suggest nutrition-related text messages involving changing Mexican food staples may have low acceptability in this population. Quantitative results showed that messages with the lowest acceptability rates included those altering recipes for burritos, eggs, and changing the preparation of beverages such as coffee and *aguas frescas* (beverage made from fruits, herbs, grains, or seeds). This finding was supported by participants expressing during their exit interviews that these changes were outside of the norm and although they would potentially try the alterations, they believed these changes would not be sustainable in the long run. Future interventions should therefore consider focusing on introducing new recipes or recipe modifications that include culture-specific staple ingredients, suggest smaller alterations to traditional foods (if any), and should promote existing healthier cultural foods such as promotion of a traditional Mexican diet pattern, which has been shown to have positive cancer-related benefits and overall health outcomes [41,42,43]. Suggestions from participants for future studies included providing education on the specific health effect of the foods or the physical activity behavior, mirroring findings from a qualitative study among Hispanic cancer survivors and caregivers [40]. Including an education component in the intervention may provide an opportunity for higher intervention adherence and promote greater behavior change [44].

### 6.2. Strengths and Limitations

This study has several strengths, including a high retention rate and high participant satisfaction, utilization of a mixed methods approach to contextualize and triangulate quantitative findings through in-depth interviews, as well as granular examination of the acceptability of individual text messages, which can contribute to greater understanding. By including the specific text messages (Supplementary Tables S1–S4) that were sent as part of the pilot intervention, we strive to enhance transparency to better inform future interventions conducted in predominantly Hispanic populations of cancer survivors and their caregivers, such as those from Southern Arizona. Limitations of this study include small sample size, particularly for caregivers, which may hinder the generalizability of results for this group. Additionally, the pilot was solely conducted with women of predominately Hispanic heritage. While several men were initially recruited, all of them declined participation at the consent stage. Future efforts should focus on strategies to promote participation of male survivors and caregivers. Further, as the current study focused on intervention geared towards a predominantly Hispanic population in Southern Arizona, the majority of participants were of Mexican ancestry. Participant feedback noted the heterogeneity of the Hispanic community and recommended future interventions expand on the cultural adaptations for a broader range of diversity. Potential strategies to address this recommendation may be including recipes and food recommendations that, while may be of a particular culture, the ingredients and/or cooking methods are familiar and recognized across Hispanic backgrounds. Additionally, validated measures of behavior change for diet and exercise are needed to enhance the rigor of the current study. Particularly implementing assessment tools that are validate among diverse populations as the one presented. Moreover, participants may have been subject to social desirability responding when being polled on the usefulness of the messages. Finally, it is important to acknowledge the possibility of bias as the majority of participants were previously involved in a lifestyle intervention study for cancer survivor symptom management. Therefore, this raises the concern not only that the feasibility results of the current study may be inflated, but that direct comparison of the intricacy of a prior lifestyle intervention with the straightforwardness of the current texting-based intervention may have introduced bias resulting in overall skewed positive remarks in the qualitative evaluation of the study.

## 7. Conclusions

This study investigated the feasibility and acceptability of a lifestyle texting intervention to promote adherence to ACS nutrition and physical activity guidelines in a predominately Hispanic sample of cancer survivors and their informal caregivers, the majority of which were English speakers and had education levels of college or above. This study provides valuable information that can inform the development of future interventions for this population, particularly regarding cultural adaptations, focusing on capability and opportunity rather than motivational strategies, and content considerations for interventions focused on diet and physical activity lifestyle behaviors. Importantly, this study adds to the growing literature on remotely delivered interventions and has important implications for accessibility, scalability, and cost-effectiveness of future trials seeking to reduce the cancer burden in this population.

## Figures and Tables

**Table 1:** Demographic and health characteristics of participants in the study (*n* = 19).

Mean (SD) or *n* (%)	Cancer Survivor (*n* = 13)	Cancer Caregiver (*n* = 6)
Age, years ^a^	62.5 (12.4)	53.2 (18.5)
Sex, female ^a,b^	12 (100%)	5 (100%)
Race and ethnicity ^a^		
Hispanic (any race)	9 (75%)	5 (83%)
Non-Hispanic Black	1 (8%)	0 (0%)
Non-Hispanic White	2 (17%)	1 (17%)
Education ^c,d^		
High school or less	1 (8%)	1 (33%)
Some college	3 (25%)	2 (66%)
Bachelor’s Degree	1 (8%)	0 (0%)
Postgraduate degree	3 (25%)	0 (0%)
Income ^a,b^		
< $10,000	3 (25%)	0 (%)
>$10,000–$30,000	4 (33%)	3 (60%)
>$30,000–$50,000	2 (17%)	1 (20%)
>$50,000	3 (25%)	1 (20%)
Preferred language ^a^		
Spanish	3 (25%)	2 (33%)
English	9 (75%)	4 (67%)
Marital status ^e^		
Single	4 (36%)	0 (0%)
Married	4 (36%)	3 (50%)
Divorced	2 (18%)	3 (50%)
Widowed	1 (9%)	0 (0%)
Caregiver relationship ^a^		
Parent	--	1 (16%)
Children	--	1(16%)
Sibling	--	1 (16%)
Friend	--	2 (33%)
Other	--	1(16%)
Employment ^b,e^		
Part or full time	3 (25%)	2 (40%)
Retired	4 (33%)	2 (40%)
Disabled	4 (33%)	0 (0%)
Stay at home	0 (0%)	1 (20%)
Cancer type ^a^		
Breast	8 (67%)	--
Head or neck	1 (8%)	--
Liver	1 (8%)	--
Endometrium	1 (8%)	--
Ovarian	1 (8%)	--
Years since treatment completion ^e^	11.9 (8.4)	--
Body mass index (kg/m^2^) ^e^	29.3 (6.8)	27.7 (5.7)
Former smoker ^a^	3 (25%)	1 (17%)

^a^ Missing *n* = 1, survivor. ^b^ Missing *n* = 1, caregiver. ^c^ Missing *n* = 5 survivors. ^d^ Missing *n* = 3, caregiver. ^e^ Missing *n* = 2, survivors.

**Table 2:** Feasibility and acceptability of a two-week ACS nutrition and physical activity-related text messaging intervention.

	Cancer Survivors (*n* = 13)	Cancer Caregivers (*n* = 6)	Total Study Sample (*n* = 19)
**FEASIBILITY** **All Messages Responded, % (*n* = 26)**	**97%**	**81%**	**92%**
Nutrition messages (*n* = 13)	98%	79%	92%
Physical activity messages (*n* = 13)	97%	82%	92%
**ACCEPTABILITY** **All Messages scored as useful, % (*n* = 26)**	**90%**	**91%**	**90%**
Nutrition messages (*n* = 13)	86%	91%	87%
Physical activity messages (*n* = 13)	94%	91%	93%

## Data Availability

The data sets generated during and/or analyzed during this study are not publicly available given the dataset includes information of marginalized communities but may be available from the corresponding author on reasonable request.

## Supplementary Materials

The following supporting information can be downloaded at: https://www.mdpi.com/article/10.3390/nu15224799/s1, Table S1: Response and acceptability rates of nutrition messages by total (*N* = 8), Survivors (*n* = 6), and Caregivers (*n* = 2) participants assigned to Bank A; Table S2: Response and acceptability rates of physical activity messages by total (*N* = 8), Survivors (*n* = 6), and Caregiver (*n* = 2) participants assigned to Bank A; Table S3: Response and acceptability rates of nutrition messages by total (*N* = 11), Survivors (*n* = 7), and Caregiver (*n* = 4) participants assigned to Bank B; Table S4: Response and acceptability rates of physical activity messages by total (*N* = 11), Survivors (*n* = 7), and Caregiver (*n* = 4) participants assigned to Bank B; Table S5: Exit qualitative interview questions and themes.

## Abbreviations

ACS	American Cancer Society
US	United States
