# Supplementary material for: Feasibility and Acceptability of a Text Message Intervention to Promote Adherence to Nutrition and Physical Activity Guidelines in a Predominantly Hispanic Sample of Cancer Survivors and Their Informal Caregivers: Results from a Pilot Intervention Trial
Source: Nutrients. 2023 Nov 16;15(22):4799. doi: 10.3390/nu15224799 (PMC10675593; doi:10.3390/nu15224799)
Supplement: Supplementary file 1 [file nutrients-15-04799-s001.zip › nutrients-2679813-supplementary.pdf]

Supplementary Table S1. Response and acceptability rates of nutrition messages by total (N = 8), Survivors (n = 6), and Caregivers (n = 2) participants assigned to Bank A*.

Bank A Nutrition Messages (English)	Bank A Nutrition Messages (Spanish)	Overall Response Rate (N = 8)	Overall Acceptability Rate (N = 8)[a]	Survivor Response Rate (n = 6)	Survivor Acceptability Rate (n = 6)[b]	Caregiver Response Rate (n = 2)	Caregiver Acceptability Rate (n = 2)[c]
Keep a bag of frozen fruit in the freezer so you always have fruit on hand to make smoothies. This can be a quick on the go breakfast.	Mantenga una bolsa de fruta en el congelador para que tenga fruta a mano para hacer batidos. Esto puede ser un desayuno rápido.	88%	100%	100%	100%	50%	100%
Make a snack that travels. Use a whole wheat tortilla & peanut butter, add fruits like bananas or strawberries, and roll the tortilla up.	Haga un bocadillo rápido. Comience con tortillas de trigo integral y mantequilla de maní, agregue frutas como plátanos o fresas, y enrolle la tortilla.	88%	71%	100%	67%	50%	100%
Do you love cream in your coffee? Try getting black coffee and using low-fat or fat free milk.	¿Le gusta crema en su café? Intente tomar café solo.	100%	38%	100%	33%	50%	0%
Instead of pan frying your meats, try baking, grilling, or broiling. No oil needed!	En lugar de freír, intente hornear, o asar a la parrilla. ¡No es necesario aceite!	88%	86%	100%	83%	50%	100%
Home-brewed unsweetened iced tea is a cheap, calorie-free, and refreshing drink! Dress it up with sliced lemons or oranges.	El té helado sin azúcar casero es una bebida barata, sin calorías y refrescante. Para obtener más sabor, exprima jugo de limón o naranja.	86%	86%	100%	83%	50%	100%
Keep fruit where you can see it, like a bowl on your kitchen counter. You'll be more likely to grab a healthy snack on your way out.	Mantenga fruta en donde pueda verla, como en una canasta en la cocina. Es más probable de tomarla antes de salir de casa.	88%	100%	100%	100%	50%	100%
Make mealtimes a time-out from TV and focus on enjoying your food. If you're watching TV while you eat, you are more likely to eat more.	Concéntrense en comer su comida. Si mira televisión o usa su teléfono mientras come es probable que coma más se sienta menos satisfecho.	88%	100%	100%	100%	50%	100%
Whole grains have more vitamins, minerals and fiber to keep you satisfied longer. Try switching to wholewheat tortillas, bread and pasta.	Granos integrales tienen más vitaminas y fibra para mantenerla satisfecha más tiempo. Intente cambiar a tortillas de trigo, pan, y pasta.	88%	71%	100%	67%	50%	0%
Love eggs? Try mixing one egg and two egg whites to reduce how much fat you are eating without losing flavor.	Intente mezclar un huevo y dos claras de huevo para reducir la cantidad de grasa que está comiendo sin sacrificar el sabor.	75%	50%	83%	60%	50%	0%
Feeling tired? Apples are great substitutes for caffeinated drinks because they give a similar energy boost minus negative side effects.	¿Siente cansancio? Las manzanas pueden sustituir bebidas con cafeína porque dan energía similar sin los efectos secundarios.	88%	86%	100%	83%	50%	100%

Making healthy choices works best when prepared. Pre-make meals for the week on Sunday night. You will have healthy food ready to eat!	Prepare comidas para la semana los domingos por la noche. Así tendrá comidas saludables preparadas y listas para comer toda la semana.	88%	86%	100%	83%	50%	100%
Did you know staying hydrated can keep you from feeling hungry? Try drinking 8 glasses of water daily to fight craving & keep you full.	¿Sabía que mantenerse hidratado puede evitar que tenga hambre? Intente beber 8 vasos de agua al día para combatir los antojos.	75%	100%	83%	100%	50%	100%
Read the Nutrition Facts Label, some foods may have more than 1 serving per package. This information is at the top of the label.	¿Sabía que algunos paquetes tienen más de 1 porción? Lea la etiqueta nutricional para ver cuantas porciones hay en el contenedor.	88%	86%	100%	83%	50%	100%

[a]Acceptibility rate calculated from the total number of participants who responded to the message. [b]Acceptibility rate calculated from the total number of survivors who responded to the message. [c]Acceptibility rate calculated from the total number of caregivers who responded to the message. * For reuse please use the suggested citation for this publication.

Supplementary Table S2. Response and acceptability rates of physical activity messages by total (*N* = 8), Survivors (*n* = 6), and Caregiver (*n* = 2) participants assigned to Bank A*.

**Bank A Physical Activity Messages (English)**	**Bank A Physical Activity Messages (Spanish)**	**Overall Response Rate (*N* = 8)**	**Overall Acceptability Rate (*N* = 8)[a]**	**Survivor Response Rate (*n*=6)**	**Survivor Acceptability Rate (*n* = 6)[b]**	**Caregiver Response Rate (*n* = 2)**	**Caregiver Acceptability Rate (*n* = 2)[c]**
Multitask! Do 20 calf raises every morning while brushing your teeth to strengthen and tone your legs. https://bit.ly/2MOIefq	Haga 20 levantamientos de pantorrillas cada mañana para fortalecer y tonificar sus piernas https://bit.ly/2MOIefq	89%	100%	100%	100%	50%	100%
Playing with children/grandchildren/pets for just one hour can burn up to 246 calories, just as many calories as walking briskly.	Jugar con sus hijos, nietos, mascotas por solo una hora puede quemar hasta 246 calorías.	88%	86%	100%	83%	50%	100%
Exercise when energy levels are high. Pain & fatigue varies daily or even hourly. Take advantage of when you feel the best to exercise.	Niveles de dolor y fatiga pueden variar de un día a otro. Haga ejercicio cuando sus niveles de energía sean altos.	100%	88%	100%	100%	100%	50%
Exercise includes washing a car for 45 min, washing floors for 45 min, gardening for 30 min, and raking leaves for 30 min.	¿Sabía que limpiar y cocinar son formas de ejercicio? Lavar un carro por 45-60 min, lavar pisos por 45-60 min y rastrillar hojas por 30 min.	88%	86%	100%	83%	50%	100%
Walking can be boring without company. Invite a family member/friend to accompany you during your daily walks and it will go by quicker!	Caminar puede ser aburrido sin compañia. Invite a un familiar o amigo para que lo acompañe en sus caminatas diarias y pasará más rápido.	88%	86%	100%	83%	50%	100%

Multitask! Try walking around while talking on the phone.	Trate de caminar mientras habla por teléfono.	75%	100%	83%	100%	50%	100%
Take more steps daily! Take 1 instead of 3 loads of laundry at a time, park far from the store, dance in place and watch steps add up!	¡Haga más pasos cada día! ¡Tome una en lugar de tres cargas de ropa a la vez o estacione más lejos de la tienda!	100%	100%	100%	100%	100%	100%
Did you know that physical activity can reduce your risk of cancer, heart disease, and diabetes?	¿Sabía que la actividad física puede reducir su riesgo de cáncer, enfermedad cardíaca y diabetes?	88%	100%	100%	100%	50%	100%
Make a list or gather a group of pictures of reasons exercise can improve your life. When you need a boost, you can revisit this list.	Haga una lista de razones por las cuales el ejercicio puede mejorar su vida. Cuando necesite un impulso puede visitar esta lista.	88%	71%	100%	83%	50%	0%
Go ahead: Step away from the computer at work for 10 min and get a few steps in!	Adelante: ¡aléjese de la computadora en el trabajo durante 10 minutos y obtenga algunos pasos!	88%	100%	100%	100%	50%	100%
Try a Zumba or Yoga class for something new, to meet people and relieve stress.	Intente una clase de Zumba o Yoga para algo nuevo, para conocer gente y aliviar el estrés.	88%	71%	100%	83%	50%	0%
Going for a walk can offer a physical escape from a stressful situation and gives you the opportunity to clear your head.	Salir a caminar puede ofrecer un escape físico de una situación estresante y le da la oportunidad de despejar su mente.	75%	83%	83%	80%	50%	100%
Add balance exercises to your daily routine. Try standing on one foot while doing dishes, brushing your teeth, or talking on the phone.	Incorpore ejercicios de equilibrio en su rutina diaria. Trate de pararse sobre un pie cuando esté lavando los platos o hablando por teléfono.	86%	83%	100%	100%	50%	0%

[a]Acceptibility rate calculated from the total number of participants who responded to the message. [b]Acceptibility rate calculated from the total number of survivors who responded to the message. [c]Acceptibility rate calculated from the total number of caregivers who responded to the message. * For reuse please use the citation for this publication.

Supplementary Table S3. Response and acceptability rates of nutrition messages by total (N = 11), Survivors (n = 7), and Caregiver (n = 4) participants assigned to Bank B*.

Bank B Nutrition Messages (English)	Bank A Nutrition Messages (Spanish)	Overall Response Rate (N = 11)	Overall Acceptability Rate (N = 11)[a]	Survivor Response Rate (n = 7)	Survivor Acceptability Rate (n = 7)[b]	Caregiver Response Rate (n = 4)	Caregiver Acceptability Rate (n = 4)[c]
Try homemade Limonada & Horchata instead of store bought, with half the amount of sugar and no preservatives. https://danzadefogones.com/horchata-de-chufa/	Horchata es más deliciosa casera, con la mitad de la cantidad de azúcar y sin conservantes https://danzadefogones.com/horchata-de-chufa/	91%	60%	100%	57%	75%	67%
Do you drink sweetened tea/sodas? Instead try water or no calorie beverage to save 200cal and 50g of sugar for each swap you make.	¿Bebe té o refrescos con azúcar? Intente reemplazar 1 cada día con agua o una bebida sin calorías y evite 200 calorías y 50g de azúcar.	100%	91%	100%	86%	75%	100%
Try making salsa with fresh vegetables (tomatoes, onions, green chili, etc.). Fresh salsa has no preservatives & is a healthier option.	Intente hacer salsa en casa con vegetales frescos (tomates, cebollas, cilantro, etc.). De esta manera, está comiendo vegetales frescos.	100%	82%	100%	86%	100%	75%
Choose air popped popcorn as a low calorie, high fiber snack.	Elija palomitas de maíz hechas al aire como una merienda baja en calorías y alta en fibra.	91%	80%	100%	86%	100%	75%
Everyone loves Tacos! Instead of frying taco tortillas, try baking them in the oven for about 5 minutes to get a similar crispiness.	¡Todos amamos los tacos! En lugar de freír tortillas intente hornearlos durante 5 minutos para obtener una textura crujiente similar.	91%	100%	86%	100%	100%	100%
Do you love dessert? Try cutting it into fourths and eating it over the span of four days (pan dulce, flan, churros, etc.).	¿Le encanta el postre? Intente cortarlo en cuartos y comerlo en el lapso de cuatro días (pan dulce, flan, churros, etc.).	100%	91%	100%	100%	100%	75%
Refried beans can be a great side dish when they are homemade and without lard. Try using alternatives for lard, like coconut oil.	Frijoles refritos son más saludables hechos en casa y sin manteca. Intente usar alternativas de manteca, como aceite de oliva o de coco.	91%	90%	86%	100%	100%	75%
Slice up a banana and dip it in dark chocolate, then place the bananas in the freezer.	Rebane un plátano y sumérjalo en chocolate negro, luego coloque los plátanos en el congelador.	100%	100%	100%	100%	100%	100%

Most juices & sports drinks have added sugars and calories. Try adding fresh cut fruit (strawberries, pineapples, and lemons) to water.	Jugos/bebidas deportivas tienen mucha azúcar y calorías. Intente agregar fruta cortada (fresa, piña, limón) a su agua para obtener sabor.	80%	100%	100%	100%	50%	100%
Instead of making tacos on tortillas, put all of the same ingredients on leaves of lettuce.	En lugar de hacer tacos en tortillas, ponga todos los mismos ingredientes en hojas de lechuga.	100%	100%	100%	100%	100%	100%
Research shows that eating healthy can improve mood & quality of life. Start a food diary of what you eat & how it makes you feel.	Comer saludable mejora su ánimo/calidad de vida. Trate de mantener un diario de alimentos y de cómo se siente durante una semana.	100%	100%	100%	100%	100%	100%
Emotional eating can lead to weight gain. If feeling stressed/anxious take 5 deep breaths. Still hungry? Grab a crunchy veggie.	Cuando sienta estrés o ansiedad tome respiraciones profundas. Si todavía tiene hambre, tome un vegetal crujiente como zanahorias o apio.	100%	91%	100%	86%	100%	100%
Leafy greens like spinach, arugula, & kale contain vitamins, minerals & antioxidants. Try including these your salad, soup, or omelet.	¿Sabía que vegetales de hoja verde contienen vitaminas y antioxidantes, y se han asociado con una disminución del riesgo de cáncer?	91%	100%	86%	100%	100%	100%

[a]Acceptibility rate calculated from the total number of participants who responded to the message. [b]Acceptibility rate calculated from the total number of survivors who responded to the message. [c]Acceptibility rate calculated from the total number of caregivers who responded to the message. * For reuse please use the suggested citation for this publication.

Supplementary Table S4. Response and acceptability rates of physical activity messages by total (*N* = 11), Survivors (*n* = 7), and Caregiver (*n* = 4) participants assigned to Bank B*.

Bank B Physical Activity Messages (English)	Bank B Physical Activity Messages (Spanish)	Total Response Rate (*N* = 11)	Total Acceptability Rate (*N* = 11)[a]	Survivor Response Rate (*n* = 7)	Survivor Acceptability Rate (*n* = 7)[b]	Caregiver Response Rate (*n* = 4)	Caregiver Acceptability Rate (*n* = 4)[c]
Consider taking the stairs rather than the elevator whenever you can.	Considere subir las escaleras en lugar del ascensor siempre que pueda.	100%	90%	100%	100%	100%	100%
Find others in your community and start a walking group. Try going to the zoo, a new park, a museum, or in a mall/ store.	Encuentre a otros en su comunidad y comience un grupo de caminatas. Intente ir a un parque nuevo, a un museo o en un centro comercial.	100%	100%	100%	100%	100%	100%
Slow down and be patient with yourself. If you are not feeling 100% walk instead of jog, or jog instead of run.	Baje la velocidad y sea paciente con usted mismo. Si no se sientes al 100%, camine en lugar de trotar, o trote en lugar de correr.	100%	91%	100%	100%	100%	100%
Not sure where to start? Ask those around you what they like to do to stay active and ask to join them.	¿No sabe por dónde empezar? Pregúnteles a los que lo/a rodean que les gusta hacer para mantenerse activos y pida que se unan a ellos.	82%	100%	86%	100%	75%	100%
Take a moment to stretch. Lean your right ear over to your right shoulder, hold for a few breaths and then switch sides.	Tómese un momento para estirarse. Incline la oreja derecha hacia el hombro derecho, aguante unas respiraciones y luego cambie de lado.	91%	90%	86%	100%	100%	75%
Increase muscle strength with daily arm raises using dumbbells/ soup cans from your pantry as light as 5 pounds.	Puede agregar fuerza muscular al hacer levantamientos de brazos con pesas o latas livianas (5 libras)	100%	100%	100%	100%	100%	100%
Afraid of falling during exercise? Look for options like riding a stationary bike or doing seated exercises in a chair/ on the floor.	Si tiene miedo de caerse mientras hace ejercicio, busque opciones como andar en bicicleta estacionaria o hacer ejercicios sentados.	91%	70%	86%	67%	100%	75%
When watching TV try marching in place with high knees or doing jumping jacks during commercial breaks.	Cuando vea televisión, intente marchar con las rodillas altas o hacer saltos durante los recesos comerciales.	91%	100%	86%	100%	100%	100%
Explore your community! Try hiking outdoors, find a new park or bike path and get out into the sunlight and fresh air!	¡Explore su comunidad! ¡Intente caminar al aire libre, encuentre un parque o sendero para bicicletas y salga al sol y el aire fresco!	100%	100%	100%	100%	100%	100%

Walking with a friend during a lunch break will wake you up & clear your mind. A good rule is to not sit for more than 1 hour at a time.	Caminar en su hora de almuerzo aclarará su mente y lo preparará para el resto del día. Intente no sentarse por más de 1 hora a la vez.	100%	100%	100%	100%	100%	100%
Did you know that physical activity strengthens not only your heart and muscles but your bones too?	¿Sabías que la actividad física fortalece no solo su corazón y sus músculos, sino también sus huesos?	100%	100%	100%	100%	100%	100%
For a bit of encouragement to exercise, think of all the reasons why you can, instead of the excuses of why you can't.	Para un poco de ánimo para hacer ejercicio, piense en todas las razones por las que puede, en lugar de las excusas de por qué no puede.	100%	91%	100%	100%	100%	75%
To increase your steps, take more trips to and from your car when bringing groceries inside and carry one bag at a time.	Para aumentar sus pasos, tome más viajes hacia y desde su automóvil cuando lleve comestibles dentro y lleve una bolsa a la vez.	100%	100%	100%	100%	100%	100%

[a]Acceptibility rate calculated from the total number of participants who responded to the message. [b]Acceptibility rate calculated from the total number of survivors who responded to the message. [c]Acceptibility rate calculated from the total number of caregivers who responded to the message. * For reuse please use the suggested citation for this publication.

Supplementary Table S5. Exit qualitative interview questions and themes.

Deductive Thematic Category	Moderator Guide Question (English/Spanish)	Inductive Thematic	Quote Example
Texting dosage	1. At any point did the messages become annoying or burdensome? Would you liked to have received more messages?<br><br>*¿Alguna vez se sintió irritada o enfadada por los mensajes? ¿Le hubiera gustado recibir más mensajes?*<br><br>2. Did you feel that the number of messages you received per day were too many, too little, or just right for helping you change your behaviors or maintain your current healthy behaviors?<br><br>*¿Sintió que la cantidad de mensajes que recibió por día eran pocos, demasiados, o adecuados para ayudarle a cambiar su estilo de vida o mantener su salud actual?*	• Participants reported the number of text messages received per day (two) was not burdensome, they looked forward to the next message, and the quantity was adequate to promote behavior change.	*"It was good, you know? I don't think you want to be bombarded. It is almost like if you only got one it would not be enough, but two, I don't know, it seems just right"*-Participant 305a (Survivor)<br><br>*"No, I never found them a burden. I liked reading them. You know, one or two [messages] reinforced what I was doing already but it gave me some new insights." – Participant 407a (Survivor)*<br><br>*"I loved it. The tips were so interesting, and I was so interested in knowing what the next text was going to say because you know we are trying to better our way of life." -Participant 332b (Caregiver)*<br><br>*"Yeah, they were not overwhelming or anything. I kind of looked forward to them and every so often I checked to make sure I got them. But yeah, I think two a day is a good number."-Participant 301b (Caregiver)*

			"I was looking forward to them and they were never burdensome. I just had to get used to them coming. If I didn't get one at what I thought was the right time, I was looking for it so I can stay motivated." *- Participant 418a (Survivor)*
Timing of texts	1. What are your thoughts on the time of day your messages were sent?<br><br>*¿Cuál es su opinión sobre los tiempos/las horas en que se enviaron los mensajes?*	• Participants reported high satisfaction with the timing of the text messages (one in the morning, one at night) because they aligned with their daily schedules, or if they didn't, participants could read them when the time was convenient.	*"[the time I received the messages] seemed fine to me. They were sent to me at a very good time because I get up early in the morning so I didn't have any problems."* *-Participant 901a (Survivor)*<br><br>*"It was convenient, I remember them coming through not during work hours, so it was a convenient time to pause and think about stuff."* -Participant 301a (Survivor)<br><br>*"Sometimes I received the messages very early in the morning, like 8, but it was fine because I answered them when I could so there was no problem."* *-Participant 342a (Survivor)*
Texting platform satisfaction	1. How did you like communicating over text message? What did you like about it? What did you not like about it?<br><br>*¿Cómo se le hizo la comunicación por mensajes de texto? ¿Qué le gusto? ¿Qué no le gusto?*	• Participants reported satisfaction with using the texting platform to receive information about lifestyle given it was an easy and accessible way to receive information that can be accessed at any time.<br><br>• Some participants shared their preference for texting over phone calls or the internet.	*"What makes text so popular is that you can answer it in your own time per say. So, it is not as you know, a phone call comes in and you go ugh I can't take that right now. With a text, you know, it has a nice response… it is not as intensive communicating, or it is not as stressful as other forms of communications. You know, you look at it, read, it and give an answer, so it works out well. I like text."* -Participant 301a (Survivor)<br><br>*"It was perfect. You know, I want to say that this was the study that I have enjoyed the most just because I had the freedom of getting to it when I had the time, or getting to it later and I never felt pressured and all the tips, very, very cool" -* Participant 332a (Survivor)<br><br>*"Although I am technologically literate, I do not have internet at home, so I do appreciate being able to get the text messages without having to go to the library and look at my email".* -Participant 301b (Caregiver)<br><br>*"I am 90 years old so doing things with cell phones is new to me. I do have to tell you, I just learned how to text and I like it!"* -Participant 422a (Survivor)<br><br>"I thought this is a great tool, this is a great way of using technology and social media to further things along." -Participant 401a (Survivor)

Texting content preferences	1. Was the information provided to you in the messages useful to you?<br><br>*¿Cree que la información de los mensajes fue útil para usted? ¿Por qué o por qué no? ¿Qué los hubieran hecho más útiles?*<br><br>2. Did you find the texts to be helpful? Do you remember the most helpful or best message you received? If so, what was it? What made this message helpful/good?<br><br>*¿Se le hicieron útiles los mensajes de texto? ¿Algún mensaje en particular que le gusto más? ¿Si es así, que mensaje fue? ¿Qué tenía este mensaje que fue útil o mejor?*<br><br>3. Think about the topics of the messages. Which topics could you relate to most? Are there any other topics you think we should consider adding?<br><br>*¿Cuáles temas le gustaron más o se le hicieron más relacionables? ¿Hay otros temas que hubiera querido ver o que usted agregaría?*	• Participants reported a preference for messages that linked the behaviors to specific biological mechanisms and health.<br><br>• Participants requested more information about how to deal with long-term cancer side effects, particularly, nausea and sleep, and the potential role of lifestyle to manage them.	*"The one I particularly liked was the one about fruit and green leafy vegetables having antioxidant and their role in cancer, as a cancer survivor, I really liked this topic"*<br>-Participant 901a (Survivor)<br><br>"I would suggest topics like foods we eat and their effects on the body. For example, when you eat sugars and it can lead to diabetes, and how diabetes can lead to kidney disease, and it is all a domino effect"<br>-Participant 325a (Survivor)<br><br>"I have nausea after my cancer and I still deal with that, so maybe other patients are dealing with it too. Maybe there can be things with how to deal with the nausea, things like have ginger tea, you know, having an extra glass of water. You know those kinds of things would be very helpful."<br>-Participant 401a (Survivor)<br><br>"One thing I find [interesting] is sleep. They can tell you to sleep but some people cannot sleep, they don't address what to do when you can't sleep."<br>-Participant 422a (Survivor)
Behavior change	1. Since receiving the messages, have you made any changes to your eating or activity (exercise)?<br><br>*Desde que empezó a recibir mensajes, ¿ha cambiado algo en su estilo de vida (actividad física o nutrición)? Si es así, ¿en qué maneras?*	• While participants reported nutrition-related messages served as good reminders of healthy eating habits, participants reported a bigger impact of the intervention on physical activity behaviors, particularly highlighting the usefulness of physical activity while multitasking.	"This whole thing was a good reminder …I think one of the reminders there was to drink a full glass of water before you eat, and I have been really good at doing that lately, but overall, this program was a good reminder too."<br>-Participant 301b (Caregiver)<br><br>"I do, as a matter of fact, there was one that helped me a lot with leg stiffness. It was the one about while you brush your teeth, get up on your tip toes…. That in itself was worth the whole thing. I have had that problem for years and now it has gone away."-Participant 402a (Survivor)<br><br>"I try to move around. like when I brush my teeth, I just bend one leg and I stand on one leg and then the other. Just doing that it makes me realize that the stiffness is there… It feels good to actually move them and the knee is getting lubricated, and you are working some muscles in the leg. And it does not take any time! "

			-Participant 305a (Survivor)<br><br>"Well, the physical activity, like I say I have tried to be conscious of multi-tasking with physical things and like I said enjoying walking and this and that and making two trips instead of one, those kinds of things."<br>-Participant 301a (Survivor)
Cultural appropriateness	1. Were the messages easy to understand and could you relate to them? How could you relate or how did you not relate to the messages?<br><br>*¿Cree que los mensajes fueron fáciles de entender y relacionables? ¿Cómo se pudo relacionar con ellos o como no se pudo relacionar con ellos?*<br><br>2. In thinking about cultural relevance, one of the goals of this study was to design messages the Latino/a community could relate to. Do you think these messages were appropriate?<br><br>*Al pensar en la relevancia cultural, el objetivo de este estudio fue diseñar mensajes con los que la comunidad Latina pudiera relacionarse. ¿Cree que estos mensajes fueron apropiados? ¿Por qué o por qué no?*	• Participants described the content to be easy to understand and universally relatable given diet and physical activity to improve health are important for everyone.<br><br>• Participants reported some hesitancy towards changing cultural food staples.<br><br>• Participants highlighted cultural differences within Hispanic subpopulations.	"The messages were concrete and I could understand them very easily, they were not long or complex, that is what I liked the most" -Participant 325a (Survivor)<br><br>"I think [the messages] were universally appropriate and when it came to food some of them were culturally relevant, but I think more than being culturally relevant, they were part of universal things."<br>-Participant 407a (Survivor)<br><br>"The one about the tacos on the lettuce leaves, the part of the tacos, the corn tortilla, is what I really like. I think I can try your suggestion, but I don't think I can sustain it"<br>-Participant 407a (Survivor)<br><br>"The ones about diet, they were great but maybe it is a cultural thing, when it says to make a burrito with peanut butter and bananas, I don't think burritos call for that. They usually have beans and cheese when you make them at home. That might not be the kind of thing I would have tried."-Participant 305a (Survivor)<br><br>"Yes and I am gonna suggest something to you if you don't mind. I am, I was born and raised in Puerto Rico, and a lot of the meals suggestions that you mentioned in the study were geared toward people with a Mexican background. You know the Mexican dishes like the tacos and the horchata…. Things like that, ummm we Puerto Ricans eat a lot of rice and beans and I don't know if something can be added geared towards other Latino communities.<br>-Participant 332a (Survivor)
